# Notification of Measles

**Published:** 1897-04-17

**Authors:** 


### Notification of Measles.

Any suggestion which gives promise of putting a check upon the wasteful loss of life which is caused by measles in this country is worthy of the most serious consideration, and we need not wonder at the interest shown in the discussion upon the notification of measles opened by Dr. Kenwood at the Sanitary Institute last week. Dr. Kenwood fully recognises the importance of the problem. During the fifteen years from 1881 to 1895, measles caused more deaths of children under five years of age in England and Wales than small-pox, scarlet fever, diphtheria, and typhoid fever combined. Yet with this fully before his eyes he declares against compulsory notification of measles, unless people are at the same time prepared to adopt much more thorough and extensive measures for controlling its outbreaks than are at all likely to be adopted by most communities.

The first and best recognised objection to the notification of measles arises from the fact that the disease is infectious for some days before its presence can be diagnosed. During this time the infection may be, and is, scattered broadcast, and whole families—and almost whole schools—may be infected before a single notification could be received.

The next great difficulty is the fact that the disease is looked on by parents so lightly that in a very large proportion of the cases they do not call a medical man. This is especially the case among the poor, among whom also the disease is especially liable to spread, and to be fatal. The result is that to be of any real service the duty of notification would have to be imposed on the parents, a proceeding which all experience shows to be productive in practice of nothing but utter failure; and if this has been the case where notification has led to no interference with the household and where failure to notify has been the outcome of mere ignorance and indifference, how much more must we expect parents to conceal the presence of the disease when its notification is found to lead to the active interference of the health officers with the domestic arrangements of the house.

This, really, is an important point; for while it is recognised that notification without further measures would be useless, the further measures demanded include not only isolation, but house-to-house visitation and inspection, disinfection of houses and of infected articles, exclusion of the rest of the family from school, and prohibition of their having personal communication with children of neighbouring houses; and also—when necessary—prohibition of those dwelling in invaded houses from carrying on their occupation. All these measures are looked upon as essential for the proper control of measles by those who advocate notification, and we must ask ourselves how far notification by the householder would ever be likely to be complete when it was once recognised that it was to be followed by such disabilities.

Then about isolation. It is said that "when a case is discovered the patient should be kept apart from other members of the family in a suitable room, and in charge of an attendant, who should have no personal communications with other inmates of the house, or should be removed to a suitable hospital." Practically this means that all the cases occurring among the poor—and not the very poor alone—must be removed to hospital, and when we consider that measles comes in great rushes about every other year, the mass of the cases being crowded into a few weeks, we can see what an enormous expenditure would be required to provide sufficient hospital accommodation for the cases, and for how large a portion of the time all this preventive machinery would be lying idle. "If notification proves a failure," says Dr. Kenwood, "it is also a costly one, and if full advantage is to be taken of the information procured by notification, an expenditure amounting to at least 6d. in the pound would be involved."

There is another point, moreover, to which Dr. Kenwood has not drawn attention, but which is a matter on which we are to a large extent in the dark, viz., what would be the condition of an adult population which, by means of notification and isolation, had been shielded from measles infection during childhood? Doubtless measles as we see it is mostly a disease of childhood, but that is to a large extent because the adult population is protected by the immunity left by the disease in early life. So far as experience teaches, it shows that measles in an unprotected population takes the form of epidemics far exceeding anything to which we are accustomed, and underlying the whole problem is this serious question:—Is not labour better spent in providing hospital treatment for such cases as require it, and thus lessening the fatality of measles, than in elaborate attempts at lessening its prevalence by means of notification and isolation, attempts which, in proportion to their success, would leave the nation liable to the disease in its epidemic form?

